# Climate of origin shapes variations in wood anatomical properties of 17 *Picea* species

**DOI:** 10.1186/s12870-024-05103-7

**Published:** 2024-05-17

**Authors:** Xiaowei Yang, Huiling Yan, Chunhui Hao, Jiwen Hu, Guijuan Yang, Sanping An, Lifang Wang, Fangqun Ouyang, Miaomiao Zhang, Junhui Wang

**Affiliations:** 1 State Key Laboratory of Forest Cultivation, Central South University of Forestry and Technology, Changsha, 410000 People’s Republic of China; 2 State Key Laboratory of Tree Genetics and Breeding, Key Laboratory of Tree Breeding and Cultivation of State Forestry Administration, Research Institute of Forestry, Chinese Academy of Forestry, Beijing, 100091 People’s Republic of China; 3 Gansu Provincial Key Laboratory of Secondary Forest Cultivation, Research Institute of Forestry of Xiaolong Mountain, Tianshui, 741022 People’s Republic of China; 4 Beijing Floriculture Engineering Technology Research Centre, Beijing Laboratory of Urban and Rural Ecological Environment, Beijing Botanical Garden, Beijing, 100093 China

**Keywords:** Spruce, Xylem anatomy, Environmental factor, Phylogenetic signal

## Abstract

**Background:**

Variations in hydraulic conductivity may arise from species-specific differences in the anatomical structure and function of the xylem, reflecting a spectrum of plant strategies along a slow-fast resource economy continuum. Spruce (*Picea* spp.), a widely distributed and highly adaptable tree species, is crucial in preventing soil erosion and enabling climate regulation. However, a comprehensive understanding of the variability in anatomical traits of stems and their underlying drivers in the *Picea* genus is currently lacking especially in a common garden.

**Results:**

We assessed 19 stem economic properties and hydraulic characteristics of 17 *Picea* species grown in a common garden in Tianshui, Gansu Province, China. Significant interspecific differences in growth and anatomical characteristics were observed among the species. Specifically, xylem hydraulic conductivity (*K*_s_) and hydraulic diameter exhibited a significant negative correlation with the thickness to span ratio (TSR), cell wall ratio, and tracheid density and a significant positive correlation with fiber length, and size of the radial tracheid. PCA revealed that the first two axes accounted for 64.40% of the variance, with PC1 reflecting the trade-off between hydraulic efficiency and mechanical support and PC2 representing the trade-off between high embolism resistance and strong pit flexibility. Regression analysis and structural equation modelling further confirmed that tracheid size positively influenced *K*_s_, whereas the traits DWT, D_r, and TSR have influenced *K*_s_ indirectly. All traits failed to show significant phylogenetic associations. Pearson’s correlation analysis demonstrated strong correlations between most traits and longitude, with the notable influence of the mean temperature during the driest quarter, annual precipitation, precipitation during the wettest quarter, and aridity index.

**Conclusions:**

Our results showed that xylem anatomical traits demonstrated considerable variability across phylogenies, consistent with the pattern of parallel sympatric radiation evolution and global diversity in spruce. By integrating the anatomical structure of the stem xylem as well as environmental factors of origin and evolutionary relationships, our findings provide novel insights into the ecological adaptations of the *Picea* genus.

**Supplementary Information:**

The online version contains supplementary material available at 10.1186/s12870-024-05103-7.

## Background

Plant stems play an essential role in mechanical support, transportation, and nutrient storage, serving as a crucial link between aboveground and belowground components and influencing water and nutrient utilization efficiency [1, 2]. The xylem is responsible for transporting water and ions from the roots into other organs and tissues [3]. In conifers, the xylem mainly comprises tracheids, xylem rays, axial parenchyma cells, epithelial cells surrounding the resin canals, and wood fibers [4, 5]. These anatomical and structural traits significantly affect the hydraulic safety efficiency, drought resistance, and growth rate of conifers [6–8]. For example, tracheids and xylem rays, the primary channels for vertical and horizontal transport, determine the embolism capacity of the xylem [9]. Similarly, as the sole channel for water transfer between adjacent tracheids, the pit also affects xylem embolisms. Compared with angiosperms, gymnosperms, especially conifers, exhibit superior water and nutrient use efficiency, enhancing their adaptability to drought and cold and, ultimately, their yield and quality [10–12]. Different tree species and individuals within the same species have undergone long-term evolution, leading to extensive variation. By modifying xylem structure and composition, they adapt to changing environments and stresses, preserving these genome variations and creating a relatively stable inheritance [13]. Currently, studies on intragenus and interspecific variations in xylem anatomical traits have been conducted in Malagasy *diospyro*s and the conifer species (*Pinaceae, Cupressaceae, Taxaceae, and Podocarpaceae*) [14]; however, the focus has primarily been on phenotypes, including basic density, water content, nutritional components, and stem functions. To date, data on genetic variation between species within a genus based on xylem traits, particularly anatomical traits remain limited.

The trade-offs among mechanical strength, wood anatomy, and water transport efficiency in coniferous species are intricate, partly due to the exponential relationships between geometry and physical properties. Song reported a negative correlation between hydraulic conductivity and tracheid cell wall thickness in 28 conifer species [15], but the similar patterns were not observed in *Eucalyptus* [16]. Generally, individuals with larger vessel sizes, halo areas, and higher amplitudes in vessel dimensions tend to exhibit higher hydraulic conductivity (*K*_s_) and lower embolism resistance (*P*_50_) values [16]. Notably, correlation analyses demonstrated that the trends in vessel size (mean and distribution) and vulnerability to cavitation were consistent at the interspecific and intraspecific levels. However, comparisons across genera, multiple changes in stem anatomy, the relationship between hydraulic conductivity and embolism resistance is not unique, and chemical alterations in the amount or type of lignin could influence the tracheids, pit size, and mechanical strength, which may lead to such a result [17, 18]. Safety and efficiency do not exhibit a trade-off because different xylem anatomical traits drive them under distinct phylogenetic controls [4]. Nevertheless, possible tradeoffs between transport efficiency and mechanical strength have been observed in co-occurring chaparral shrubs and evergreen species [19, 20]. Different growth forms and habitats contribute to “optimum” stem structural differences. The trade-off between mechanical strength, conductive efficiency, and wood anatomy remains highly debated and uncertain. Multispecies studies sometimes shed light on trends within a given genus, emphasizing the need to determine whether observed trends at the genus level are applicable at the intraspecific level. The variations in xylem hydraulic and mechanical properties associated with stem dimensions and their potential impacts on plant growth remain unclear. Understanding the trade-off between mechanical strength, conductive efficiency, and wood anatomy is vital for predicting the responses of species to climate change in natural environments [21]. This knowledge is also essential for phenotyping purposes in genetic improvement programs for forest species to select genotypes better adapted to abiotic stresses [22]. Therefore, in this study, we compared species within the same genus cultivated at the same location to minimize phylogenetic biases and differences in environmental factors that could affect trait plasticity. The stem growth of conifer species was determined by pit aperture size and hydraulic tracheid diameter [15].

The intricate interplay of multiple traits shapes plant growth, development, and adaptability to the environment. Among these, xylem anatomical traits exhibit covariation, synergistically regulating xylem function and consequently affecting tree growth, fitness, and lifespan. Mechanical and tracheid-related properties are key traits involved in this process. The anatomical and structural characteristics of coniferous trees play vital roles in their responses to abiotic stress, reflecting the phenomenon of “embolism resistance” in plants [23]. Understanding the coupling relationship of xylem anatomy in conifers is pivotal for assessing their hydraulic safety efficiency during periods of drought. Notably, research has consistently highlighted the presence of synergies and tradeoffs among various anatomical traits in the xylem; for instance, a significant negative correlation was observed between tracheid diameter and hydraulic diameter, whereas the wall-to-cavity ratio exhibited a significant positive correlation with double-wall thickness [8]. Tracheid and pit traits displayed a notable positive correlation [15]. These insights expand our understanding of the diverse growth strategies adopted by conifers; when plants are subjected to environmental stress, they need to adjust their growth strategies to maximize the use of available resources and maintain survival.

Specific anatomical traits show significant positive correlations with tree height, indicating their influence on plant growth. Wide tracheids and pits promote plant growth, whereas the proportions of earlywood and latewood directly affect hydraulic security and efficiency [7]. Moreover, the tangential and chord diameters of earlywood tracheids are significantly correlated with tree height [24]. Furthermore, double-wall thickness is a crucial factor affecting the quality and strength of wood. Although some studies have been conducted on oak trees [25], there remains a relative dearth of research on the anatomical traits of coniferous trees and their relationships with tree growth traits. Consequently, this study aimed to fill this knowledge gap and shed light on the crucial interplay between the anatomical traits of coniferous trees and their coupling relationship. Understanding these relationships will expand our understanding of plant physiology and facilitate the formulation of effective strategies for managing and conserving coniferous tree species.

Phenotypic plasticity represents the diverse phenotypes plants exhibit in response to environmental fluctuations and is a crucial ecological strategy that enables their adaptation to heterogeneous environments [26]. However, despite the influence of genetics, the legacy effects of provenance environments persist in plants, even in new habitats. Notably, studies have demonstrated that the climate of origin can significantly affect xylem anatomical traits, playing a pivotal role in shaping the external expression of plants [27]. Ren has highlighted the association between provenance latitude and variation in functional traits in the *Phragmites australis* [26], showing its potential effect on phenotypic plasticity. Similarly, investigations on *Fagus sylvatica* and *Quercus* revealed altitude-dependent variations in xylem anatomical traits [7, 28]. However, although the anatomical traits of *Quercus* wood rely primarily on the environment of origin and exhibit limited plasticity across domains, in *Pinus taeda*, both mean annual temperature (MAT) and mean annual precipitation (MAP) affect xylem anatomy. Conversely, a study on *Picea abies* has shown that the pits of the xylem remained unchanged despite changes in altitude [24]. Similarly, Song investigated the anatomical structure of the xylem in 28 coniferous trees and found that the environment of origin had a minimal impact on pits and tracheids. This apparent discrepancy suggests that the influence of the climate of origin on xylem anatomy varies depending on the species, the extent of environmental variation, and the degree of trait plasticity. Given these contrasting findings, it is important to investigate the potential relationships between stem anatomical traits and climatic factors, particularly in coniferous species. The results of this study will facilitate improved forestry management practices and enhanced success of cross-regional introduction and promotion initiatives.

Herein, we focused on the *Picea* genus as the model taxon to advance our understanding of this domain. Spruce, the third-largest genus in the Pinaceae family, comprises approximately 34 species. Spruce species are widely distributed in the Northern Hemisphere and play a significant role as part of the coniferous flora in this region. This species is characterized by straight trunks and valuable wood, resulting in extensive use. Among these species, 8 occur in the United States, 2 in Europe, and 24 in Asia. Several studies have investigated the differences among various spruce species, primarily concentrated on phenotypic traits, such as wood mechanical properties, needle dimensions, and photosynthetic characteristics. However, studies focusing specifically on wood anatomy are relatively scarce. Only Ouyang et al. examined the anatomical properties of 17 spruce species, concentrating solely on traits like ring width, tracheid length and width, but overlooked certain traits associated with hydraulic transport, such as pits and hydraulic diameter [29]. This scarcity can be attributed to the challenges posed by the wide distribution range of spruce, which makes it difficult to collect representative samples and significantly impedes research in this area. To address these knowledge, our study aimed to overcome interspecific differences arising from heterogeneous environments by collecting and planting 17 spruce species with origins spanning various arid and cold regions at the same experimental site. This study encompassed the analysis of 19 xylem anatomical traits to (1) compare growth traits and wood anatomical differences among different spruce species; (2) explore correlations among various anatomical traits and investigate the influence of key anatomical and mechanical traits on growth characteristics; (3) investigate the legacy effect of provenance on anatomical traits after removing differences arising from later cultivation environments. We used an in-depth analysis of the differences in xylem structure among *Picea* species to elucidate trade-off relationships between traits, explore their impact on growth, and determine the legacy effect of provenance. These findings can potentially inform effective strategies for the sustainable management and conservation of these important tree species. Understanding the intricate interplay between climatic influences and xylem anatomical traits in conifers will advance our understanding of plant physiology and aid in developing more effective strategies for the sustainable management and conservation of valuable tree species.

## Methods

### Study site and field experiments

**Fig. 1:**
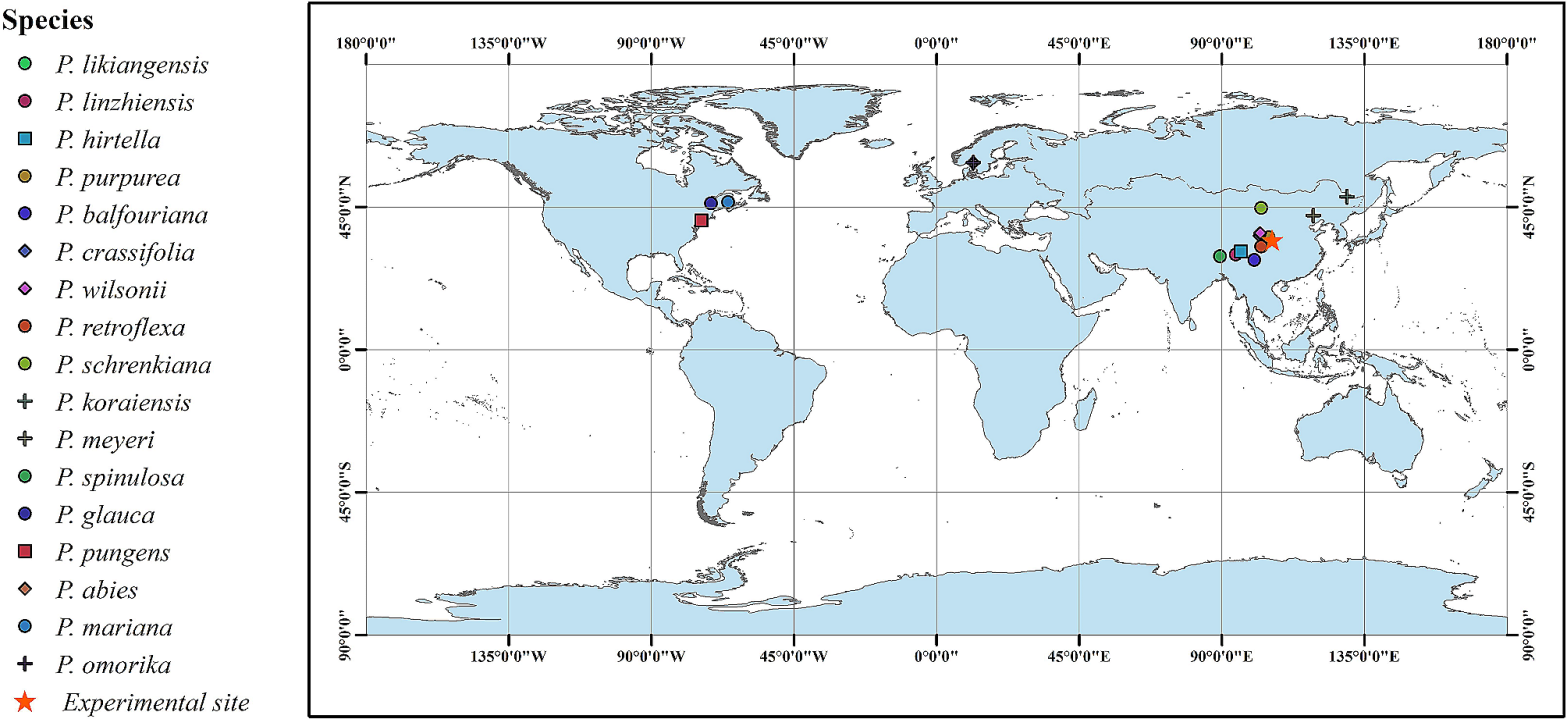
Collection sites of the 17 *Picea* species. For simplicity, the species point distributions use the average latitude and longitude for all source locations. Point shapes and colours distinguish among species

We conducted our study in a common garden at the Research Institute of Forestry of Xiaolong Mountain (34°28′50ʺN, 105°54′37ʺE) in Gansu Province, China in 2014. Within this region, the mean annual temperature is 10.7 °C; maximum annual temperature 40 °C; minimum annual temperature − 19.2 °C; mean annual rainfall 700 mm; annual precipitation 600–800 mm; altitude is 1,160 m a.s.l [29]. . This study was conducted in a common garden experiment where 17 *Picea* species were planted in 2011. Among them, *P. glauca*, *P. pungens*, *P. abies*, *P mariana*, and *P. omorika* were induced overseas. The seeds were sown in 2008, and the seedlings were planted in the common garden with a spacing of 1.5 m ×1.5 m after 3 years of cultivation. Such a common garden experiment allows the comparison of the performance of different species under similar climatic and soil conditions, thereby correcting for potentially confounding phenotypic responses to environmental variation. Meteorological data for the experimental site and the geographic sources of the 17 *Picea* species were collected by reference [30] and the Global Biodiversity Information Facility (GBIF) (http://www.gbif.org) (Table S1, Fig. 1). Ten healthy individuals without pests were randomly selected, and destructive methods were used to obtain samples. Two adjacent 2–3 cm chain-sawed disks from each tree were collected from the base of the main stem and used for measurements in 2014, and 19 wood anatomical properties were measured (Table 1).

**Table 1:** Overview of traits, the abbreviations, trait category and units as measured and used for 17 *Picea* genus in Tianshui, Gansu

Trait category	Abbreviation	Anatomical Features Description	Units
Mechanics	DWT	Double wall thickness	µm
	CWR	Cell wall ratio	%
	NR	Number of rays	mm^− 2^
	FL	Fiber length	µm
	TSR	Thickness to span ratio	-
	RH	Ray height	µm
Tracheid traits	D_r	Tracheid diameter(radial)	µm
	LD_r	Tracheid lumen diameter(radial)	µm
	D_c	Tracheid diamete(chordwis)	µm
	LD_c	Tracheid lumen diameter(chordwis)	µm
	TD	Tracheid density	mm^− 2^
	D_h_	Hydraulic diameter(theory)	kg m^− 1^ s^− 1^
	Ks	Xylem hydraulic conductivity	kg m^− 1^ Mpa^− 1^ s^− 1^
Pit traits	DPM	Pit membrane diameter	µm
	DT	Torus diameter	µm
	DPA	Pit aperture diameter	µm
	TO	Torus overlap	-
	MF	Margo flexibility	-
	VE	Valve effect	-

### Mechanical traits and tracheid dimensions

The wood samples were cut into 10.00–15.00 μm cross-sections with diagonal serial sections from pith to bark with a wood slicing machine (1400 Slip type; Leitz, Wetzlar, Germany). After the cross-sections were dried and gradually dehydrated with ethanol, the edges of the coverslips were sealed with rubber cement. Tracheid traits were determined using the nitric and chromic acid mixture segregation Tayler staining method. Six mechanical characteristics and six tracheid traits were measured or calculated for the 6-year ring. Notably, for fiber length, we randomly selected 50 tracheids from the early wood of the sixth ring of the cross-section, calculated the average value as the fiber length (FL), and simultaneously measured the double-wall thickness (DWT) to calculate the cell wall ratio (CWR). In addition, we observed the number of wood rays (NR) per unit area on the cross-section. The heights of five wood rays on the radial section were counted, and the average value was calculated as the wood ray height (RH). However, 50 tracheids were measured to calculated tracheid diameter (radial) (D_r), tracheid lumen diameter(radial) (LD_r), tracheid diameter (chordwise) (D_c), and tracheid lumen diameter (chordwise) (LD_c). We also counted the number of tracheids per unit area (TD). They were only measured for the earlywood in the sixth ring because the earlywood has the strongest effect on the embolism resistance of plants [31]. All measurements were performed using ImageJ software. The mean hydraulic diameter (*D*_h_) was calculated according to Sperry and Hacke (2004) as Σdt^5^/Σdt^4^, where dt is the diameter of each analysed tracheid. Potential hydraulic conductivity (*K*_s_) was calculated according to the Hagen-Poiseuille law:

*K*_s_*=*(πρ_*w*_/128η)×*CD*×*D*^4^ (1).

Where *K*_s_ is the potential specific stem conductivity (in kg m^− 1^ Mpa^− 1^ s^− 1^), η is the viscosity of water at 20 °C (1.002 × 10^− 3^ Pa s at 20 °C), *ρ*_*w*_ is the density of water at 20 °C (998.2 kg m^− 3^ at 20 °C), CD is the TD, and D is the hydraulic diameter (in m).

### Pit traits

**Fig. 2:**
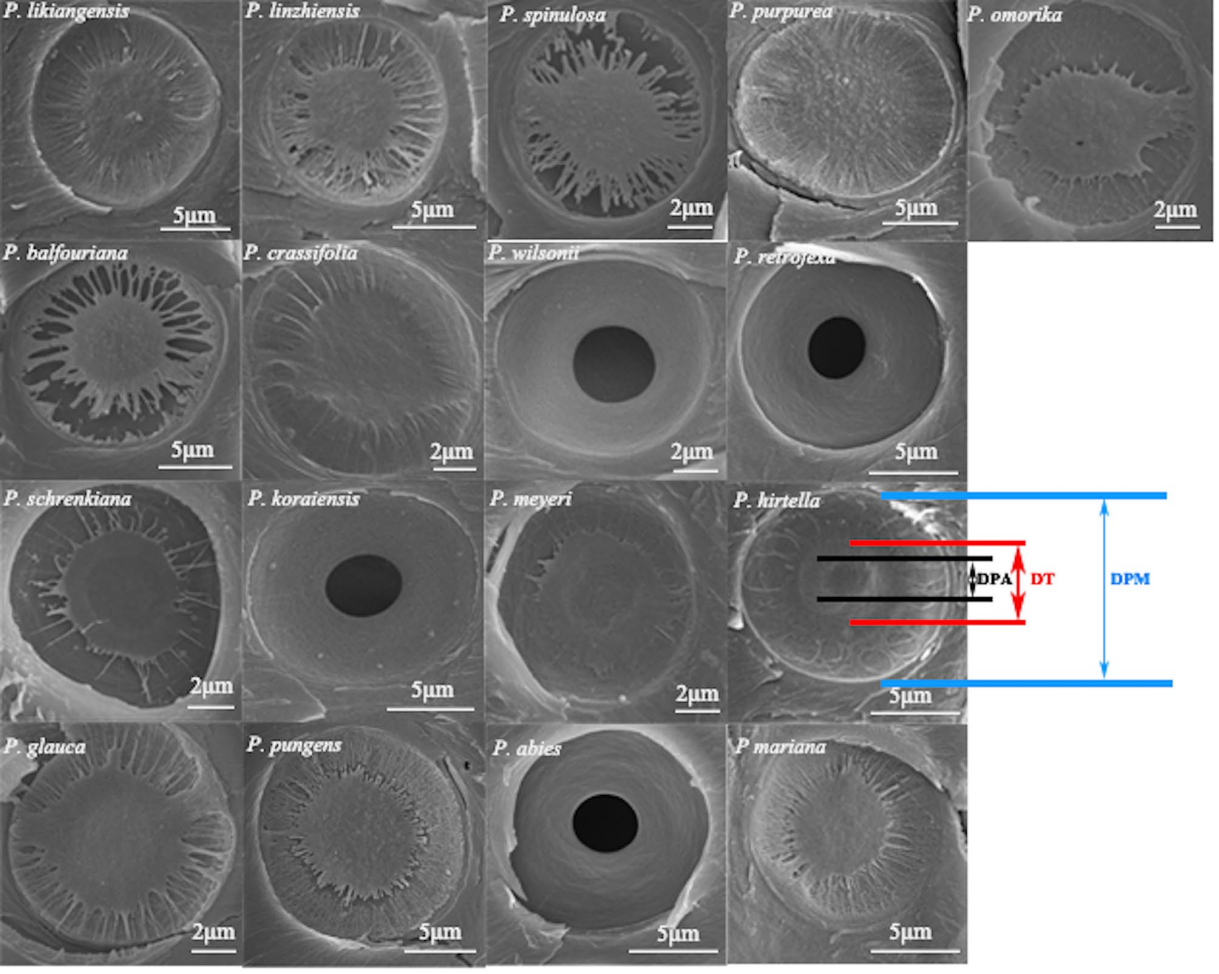
Scanning electron microscopy images of pit structure in the earlywood of 17 *Picea* species, illustrating the variation in pit size. Different pit traits are indicated with different colours. DPM, pit membrane diameter (blue); DT, torus diameter (red); DPA, pit aperture diameter (black)

For the analyses of the pit architecture, core segments 2–3 cm in length were transversally cut with a microtome (Sledge Microtome G.S.L. 1, Schenkung Dapples) and dehydrated for 3 d in an oven (80 °C). All specimens were mounted on aluminium stubs and coated with gold using a tabletop sputtering device (Leica EM SCD050, Leica Microsystem, Wetzlar, Germany). We successively observed the characteristics of the pits on the tracheids in the earlywood of the sixth ring and obtained five traits. These consisted of pit membrane diameter (DPM) and aperture diameter (DPA) of the tracheid, where each sample was observed in two directions (the chord and radial direction), and each direction was randomly observed in four complete pits. Figure 2 shows some characteristics of tracheid pits in 17 species of spruce. The pits were observed using a scanning electron microscope (model XL 20, Philips, Amsterdam, Netherlands). To assess the sealing function of the torus in embolism resistance, three anatomical traits were calculated according to [32]: margin flexibility (MF), torus overlap (TO), and valve effect (VE). MF, TO, and VE were calculated as follows:

TO = (DT-DPA) / DT (2).

MF = (DPM-DT) / DPM (3).

VE = TO×MF (4).

### Data analysis

Differences between species were evaluated using a one-way analysis of variance (ANOVA) with Tukey (HSD) post-hoc tests. Before analysis, the data were logarithmically transformed to improve normality and homoscedasticity. We used the absolute values of the Pearson’s correlation coefficients to perform a cluster analysis. Pearson’s correlation and cluster analyses were performed using the R packages HMISC and PHEATMAP, respectively. Second, principal component analysis (PCA) was performed using CANOCO 5 based on the identified cluster groups. We used the first two principal components because the third and fourth PC axes explained considerably less variation. Structural equation models (SEMs) were used to identify the direct and indirect effects on hydraulic conductivity. To compare effect sizes, all data were standardized before analysis by subtracting the mean from the trait values and dividing by the SD. The model was accepted when the P-value of the χ^2^ statistic was > 0.05. SEMs were built using the R package ‘PiecewiseSEM’ (Lefcheck J S. piecewiseSEM: Piecewise structural equation modelling in r for ecology, evolution, and systematics. Pearson correlations were calculated to assess how climatic harshness of origin affected stem and height growth and the underlying functional traits. All data analyses were performed using R v.4.0.2. To estimate the phylogenetic signals of anatomical traits of the wood, we calculated Blomberg’s K [33] using the most recent phylogenetic tree published for the *Picea* genus [34]. In addition, we used Pagel’s λ, a scaling parameter for phylogeny that measures phylogenetic dependence of observed traits data based on likelihood optimization [35].

## Results

### Interspecific and intraspecific variation of growth traits of 17 *Picea* species

**Fig. 3:**
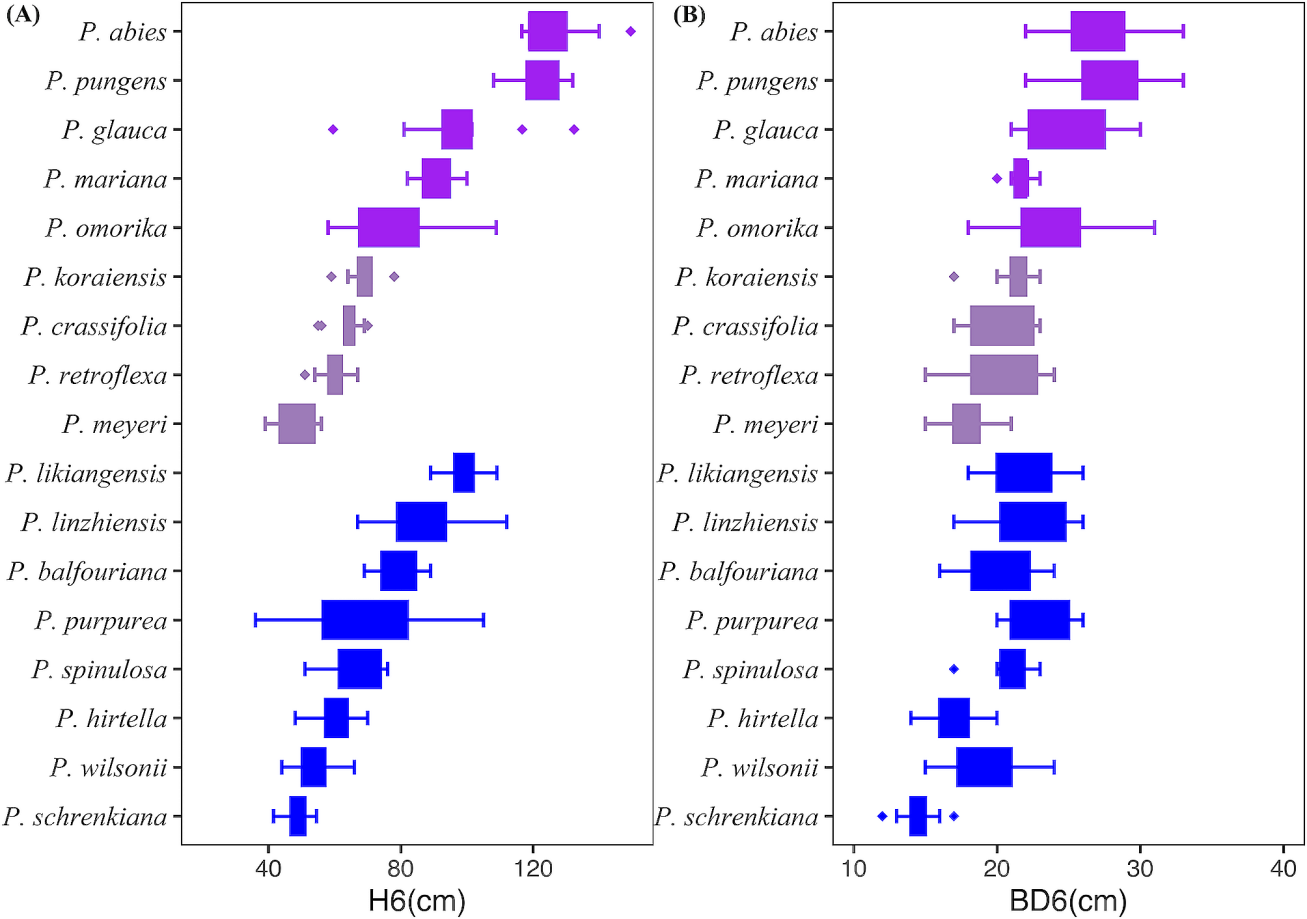
The tree height (*H*_6_) and the basal diameters (*BD*_6_) at six years old of the 17 *Picea* genus

There were extremely significant differences in tree height (*H*_6_) among the species (*P* < 0.0001), and the range of variation between species was relatively large (36–156 cm), with an average tree height of 78.34 cm (Fig. 3A). *P. abies*, *P. pungens*, and *P. glauca* grew much faster than the average growth rate. *P. meyeri* and *P. schrenkiana* were the slowest, and the growth rate of *P. abies* was almost three times that of *P. meyeri.* Of the 12 domestic spruce species, only *P. likiangensis* had a tree height exceeding 100 cm, while the *H*_6_ of the other 11 species was concentrated between 50 and 70 cm. *P. omorika* grew relatively slowly among the five foreign spruces, but it was also taller than the overall average. In summary, foreign spruce grew faster than domestic, demonstrating good environmental adaptability. We also observed that domestic spruce had small intraspecific differences and the most stable growth among individuals. Most of the 17 spruce species had a ground diameter of approximately 2 cm, still, the differences among species were highly significant (*P* < 0.0001), and the basal diameters (*BD*_6_) of *P. meyeri* and *P. schrenkiana* were relatively small, showing poor environmental adaptability (Fig. 3B).

### Comparative analysis of anatomical traits of wood

Stem anatomical traits exhibited varying levels of interspecific and intraspecific variability across 17 genus (Table 2, Table S2). The overall variation level was 3.75–58.67%, among which *K*_s_ had the largest interspecific variation (58.67%), *P. purpurea* had the largest *K*_s_, while *P. schrenkiana* had the smallest; followed by TD (23.90%), the number of tracheids per unit area of *P. schrenkiana* was the most, and *P. purpurea* was the opposite; the variation of MF was the smallest (3.75%), the MF of *P. spinulosa* was the smallest, and that of *P. omorika* was the largest. Among the 19 traits, 7 whose interspecific variation exceeded 10%, accounting for 36.84% of the total traits. The variation level of mechanical traits and pit traits was relatively stable, with a coefficient of variation between 3.75% and 12.30%. In contrast, the variation level of tracheid traits was found to be unstable at 58.67%, represented by *K*_s_, with the lowest being 7.04% (Table S2). Stem functional traits exhibit extremely significant interspecific variation (Table S2, *P* < 0.001).

**Table 2:** Differences in stem anatomical traits of 17 *Picea* species

Species	Pit traits			Trachied traits				Mechanical traits	
	DT (µm)	DPA (µm)	DPM (µm)	D_c (µm)	LD_c (µm)	LD_r (µm)	D_r (µm)	TSR	CWR (%)
*P. abies*	5.86 ± 0.52d	3.13 ± 0.31cde	11.32 ± 1.27defg	19.30 ± 2.37cde	14.68 ± 2.66bcde	20.39 ± 4.27a	23.35 ± 3.60ab	0.56 ± 0.13defg	40.56 ± 5.96cdef
*P. balfouriana*	7.07 ± 0.64a	4.16 ± 0.61a	12.89 ± 0.91ab	19.49 ± 1.94 cd	15.00 ± 1.82bcd	17.09 ± 1.74cde	21.61 ± 1.52 cd	0.58 ± 0.12defg	39.83 ± 6.28def
*P. crassifolia*	7.00 ± 0.34a	4.16 ± 0.27a	12.68 ± 1.16ab	17.87 ± 1.35defg	14.13 ± 2.41cdef	17.26 ± 1.53 cd	21.66 ± 1.75 cd	0.61 ± 0.08cdef	42.38 ± 6.89bcde
*P. glauca*	5.86 ± 0.25d	3.31 ± 0.10bcd	10.95 ± 0.57 fg	17.97 ± 0.71defg	13.69 ± 0.91def	16.60 ± 1.30cdef	21.38 ± 1.47cde	0.63 ± 0.06bcd	43.40 ± 3.79abcd
*P. hirtella*	5.79 ± 0.42d	3.02 ± 0.46de	11.34 ± 0.66cdefg	18.63 ± 2.70cdefg	14.03 ± 2.53cdef	13.50 ± 1.20ij	18.07 ± 1.26gh	0.68 ± 0.13ab	44.60 ± 4.55ab
*P. koraiensis*	7.02 ± 0.79a	4.12 ± 0.60a	12.81 ± 1.30ab	18.64 ± 1.56cdefg	14.34 ± 1.45cdef	16.14 ± 1.46def	20.44 ± 1.31de	0.56 ± 0.05efg	39.12 ± 1.51ef
*P. likiangensis*	6.25 ± 0.62 cd	3.23 ± 0.43bcd	12.27 ± 1.17abcd	21.47 ± 2.69ab	16.80 ± 2.31a	18.00 ± 2.23bc	22.67 ± 2.54abc	0.54 ± 0.06 fg	39.43 ± 5.12ef
*P. linzhiensis*	6.40 ± 0.38bc	3.41 ± 0.17bcd	12.38 ± 1.01abc	20.18 ± 1.64bc	15.20 ± 2.30bcd	16.78 ± 1.34cdef	22.08 ± 1.03bc	0.63 ± 0.09bcd	43.11 ± 5.28abcd
*P. mariana*	6.05 ± 0.51 cd	3.12 ± 0.34cde	11.93 ± 0.99bcdef	17.62 ± 1.48 fg	13.59 ± 1.35def	14.45 ± 2.15ghi	18.63 ± 2.04fgh	0.58 ± 0.08def	42.02 ± 4.89bcde
*P. meyeri*	7.15 ± 0.22a	4.14 ± 0.44a	13.17 ± 0.46a	17.56 ± 1.27 fg	13.30 ± 1.37ef	14.29 ± 1.89hi	18.84 ± 1.93 fg	0.67 ± 0.10abc	43.54 ± 4.02abc
*P. omorika*	5.82 ± 0.46d	2.80 ± 0.24e	11.87 ± 1.01bcdef	21.97 ± 5.85a	17.04 ± 5.106a	18.87 ± 3.43b	23.69 ± 4.08a	0.62 ± 0.13bcde	45.42 ± 5.63ab
*P. pungens*	6.48 ± 0.38bc	3.55 ± 0.56b	12.34 ± 1.01abcd	18.13 ± 0.08defg	14.25 ± 1.77cdef	15.79 ± 1.12efg	20.09 ± 1.02ef	0.58 ± 0.07defg	44.48 ± 0.77ab
*P. purpurea*	5.91 ± 0.66d	3.28 ± 0.39bcd	11.17 ± 1.44efg	20.04 ± 2.50bc	15.72 ± 2.28abc	17.90 ± 1.90bc	22.39 ± 1.59abc	0.52 ± 0.07 g	37.17 ± 5.45f
*P. retroflexa*	7.07 ± 0.34a	4.13 ± 0.27a	12.94 ± 0.84ab	17.70 ± 1.32efg	13.06 ± 1.57ef	15.60 ± 0.23fgh	19.96 ± 2.38ef	0.68 ± 0.10ab	44.99 ± 6.17ab
*P. schrenkiana*	6.44 ± 0.47bc	3.56 ± 0.51b	12.19 ± 0.62abcde	17.13 ± 2.00 g	12.71 ± 2.08f	12.90 ± 1.44j	17.36 ± 0.35 h	0.70 ± 0.11a	46.37 ± 6.29a
*P. spinulosa*	5.85 ± 0.50d	3.46 ± 0.47bc	10.64 ± 0.87 g	18.99 ± 0.14cdef	16.05 ± 2.70ab	14.71 ± 2.01ghi	18.91 ± 2.26 fg	0.60 ± 0.07def	42.74 ± 4.93abcde
*P. wilsonii*	6.73 ± 0.50ab	3.63 ± 0.25b	12.94 ± 1.53ab	18.84 ± 1.85cdef	14.53 ± 2.00bcde	14.53 ± 2.01ghi	18.84 ± 2.18 fg	0.62 ± 0.08bcde	44.26 ± 4.64ab
*P-value*	0.001	0.001	0.001	0.001	0.001	0.001	0.001	0.001	0.001

Stem anatomical traits were expressed as the mean ± standard deviation. All *Picea* spp. were 6 years old. ANOVA of stem anatomical characteristics among the 17 *Picea* species

### Trait associations

**Fig. 4:**
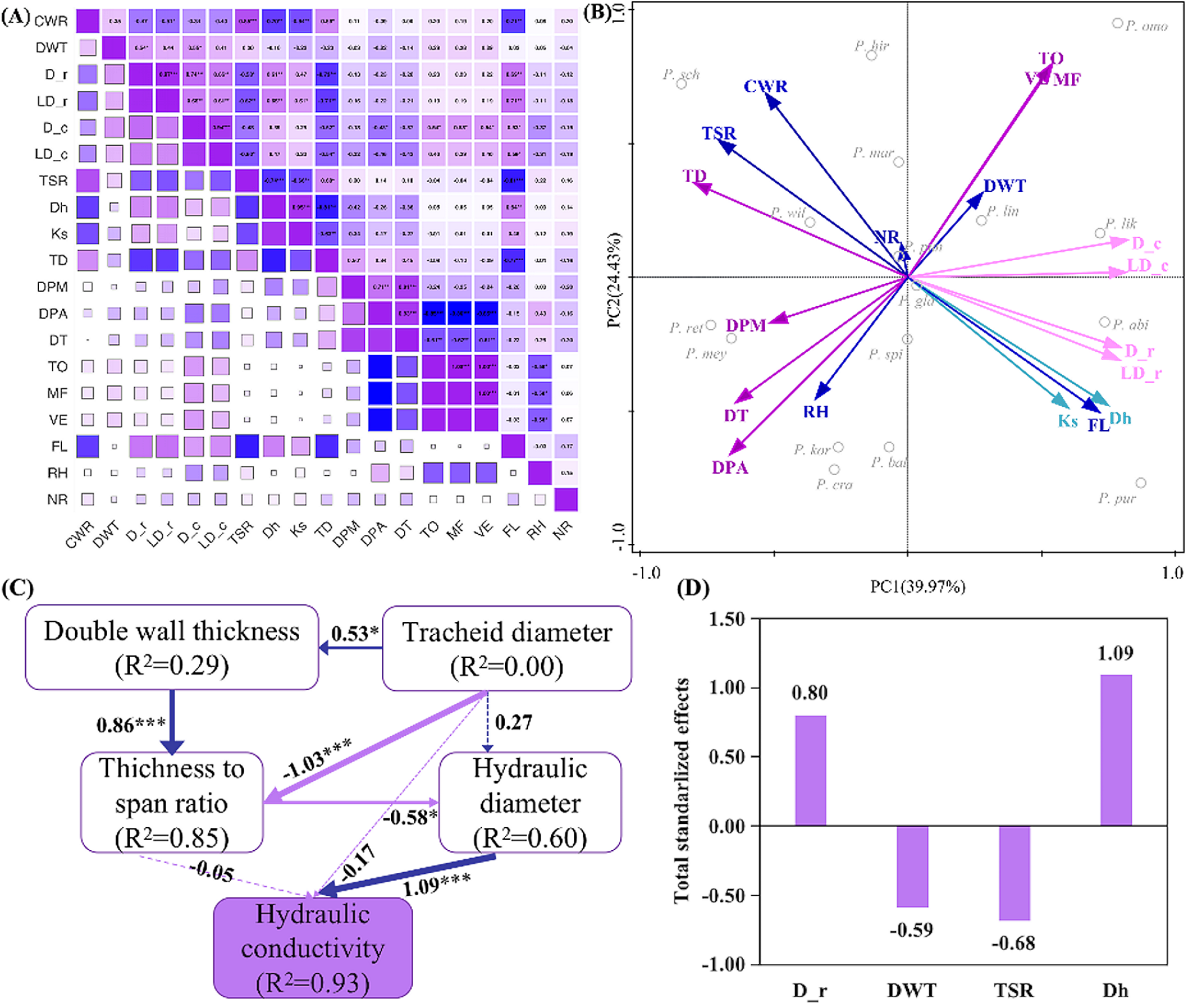
(**A**) Correlations among bark anatomical traits of 17 *Picea* species. *P < 0.05, **P < 0.01, ***P < 0.001. Blue represents negative correlation, and purple represents positive correlation. The stronger the correlation, the darker the colour, and the greater the absolute value of the number in the corresponding square. For trait abbreviations, see Table 1; (**B**) PCA of multivariate trait associations across 17 *Picea* species. The first two PCA axes and the loadings of 19 traits are shown. Different trait groups are indicated with different coloured arrows (pit traits, purple; tracheid traits, pink; hydraulic traits, lake blue; mechanics traits, blue). For trait abbreviations, see Table 1; for species abbreviations (in grey), see Supplementary Table S1. (**C**) SEM shows the relationships among the hydraulic conductivity, tracheid diameter (only tracheid diameter(racial) was used), double wall thickness, thickness to span ratio, hydraulic diameter. The structural equation diagram indicates the direct effects between two variables. The blue arrows represent positive effects, while the purple ones represent negative effects. The asterisk indicates that the effects are significant (*P < 0.05, **P < 0.01, ***P < 0.001). The dotted lines indicate that these effects are not significant. (**D**) Panels represent standardized total effects (direct plus indirect effects) derived from the SEM model, for trait abbreviations, see Table 1

The heatmap of the 19 traits showed that wider tracheids increased hydraulic conductivity and water supply. Therefore, longer fibers were required to maintain the stability of the xylem, at the expense of reducing the number of tracheids per unit area (Fig. 4A). Pit-related traits were stronger in pairs and were barely affected by the other characteristics. The TSR significantly affected wood anatomical traits and was negatively correlated with *D*_h_ and *K*_s_. In conclusion, the relationships between xylem anatomical traits were intricate, demonstrating a trade-off between water use and survival.

We used PCA to identify the major hydraulic spectra and tradeoffs. The first two PCA axes explained 64.40% of the variation (Fig. 4B). The PC1 axis reflected the trade-off between hydraulic efficiency (wide LD, D), hydraulic conductivity, and strong mechanical support. The PC2 axis reflected a trade-off between high embolism resistance (small pit DPA, DPM, and DT) and strong pit sealing (high To and VE), and wide pit dimensions for *P. ret, P. mey, P. spi, P, kor*, and *P. cra*, which had small LD, D, and *K*_s_ but large TD, thereby improving water transportation efficiency by increasing water transportation channels. In addition, *P. omo, P. lik, P. lin, P. abi, P. pur*, and *P. gla* had wide LD with MF and greater FL to prevent embolisms. SEM proved that the influence of both DWT and D_r on *K*_s_ was mediated through their impact on TSR, which subsequently affected *D*_h_, ultimately impacting hydraulic conductivity (Fig. 4C). The total standardized effects of DWT, D_r and TSR on *K*_s_ were − 0.59, 0.80, -0.68, respectively (Fig. 4D).

### Effects of pit and tracheid characteristics on growth and *K*_s_

**Fig. 5:**
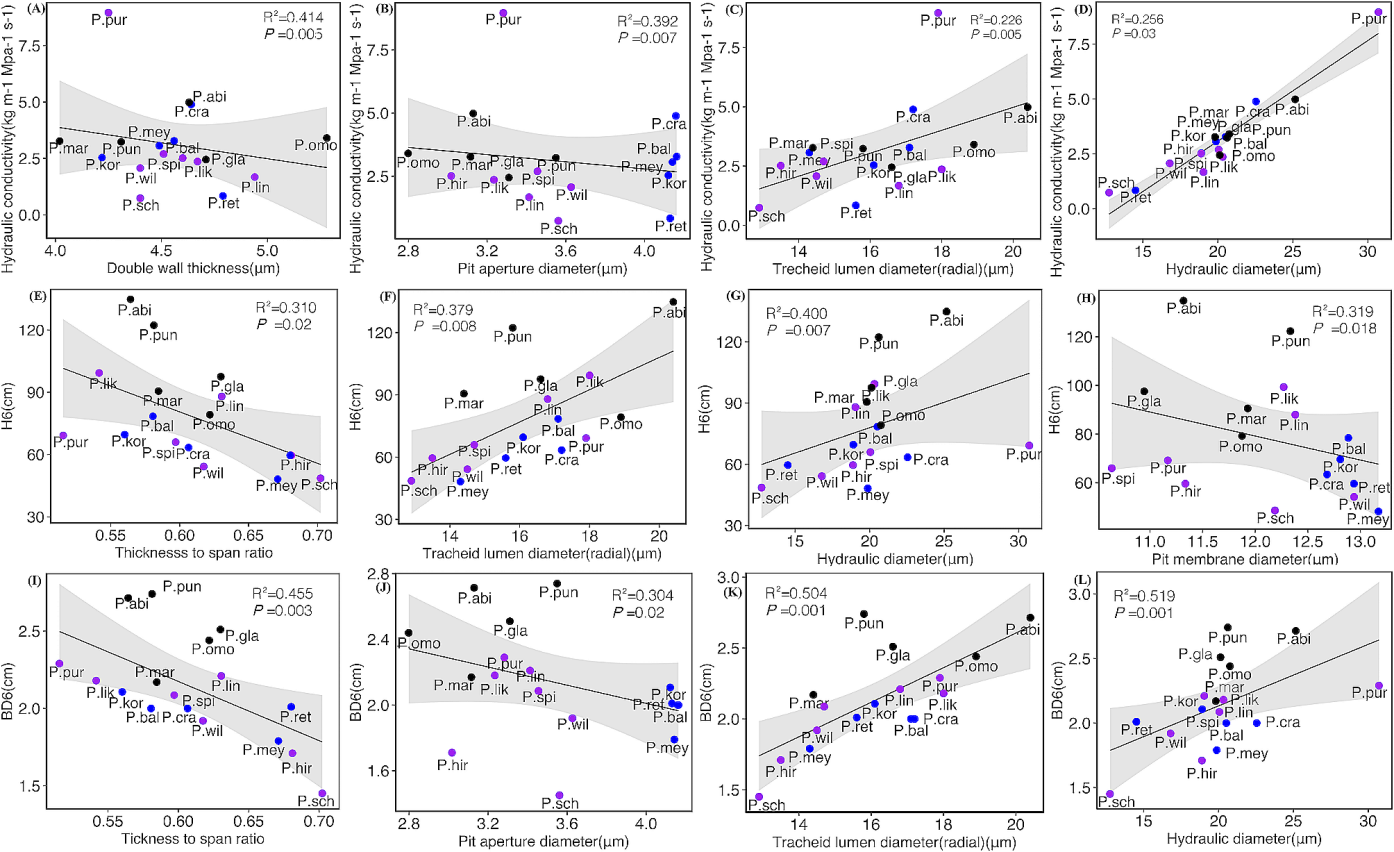
Bivariate relationships between hydraulic conductivity (*K*_s_), *H*_*6*_, *BD*_*6*_, and underlying properties for 17 *Picea* species. **A**-**D** The relationship between *Ks* and double wall thickness, pit aperture diameter, tracheid lumen diameter (radial), hydraulic diameter; **E**-**H** The relationship between *H*_*6*_ and thickness to span ration, tracheid lumen diameter (radial), hydraulic diameter, pit membrane diameter; **I**-**L** The relationship between *BD*_6_ and thickness to span ratio, pit aperture diameter, tracheid lumen diameter (radial), hydraulic diameter. Bivariate error bars (± SE of the mean), regression lines, 95% confidence intervals (grey), coefficients of determination (R^2^), and P-values are shown

To explore the effect of wood anatomical traits on *H*_6_, *BD*_6_, and *K*_s_, we first conducted a fitting analysis between different traits and *H*_6_, *BD*_6_, and *K*_s_ (Fig. 5, Fig. S1-S3). Both LD_r and *D*_h_ were positively correlated with *H*_6_, *BD*_6_, and *K*_s_. In contrast, the DPM and TSR were negatively associated with *H*_6_, *BD*_6_, and *K*_s_ (Fig. 5, Fig. S1-S3). Furthermore, we also observed that with the increase of DPA, *BD*_6_ and *K*_s_ decreased instead, and with the increase of D_c, *BD*_6_ and *K*_s_ also increased. Double wall thickness and torus diameter were significantly negatively correlated with *K*_s_ (Fig. 5A, Fig. S3K).

### Phylogenetic correlations and phylogenetic signals

**Fig. 6:**
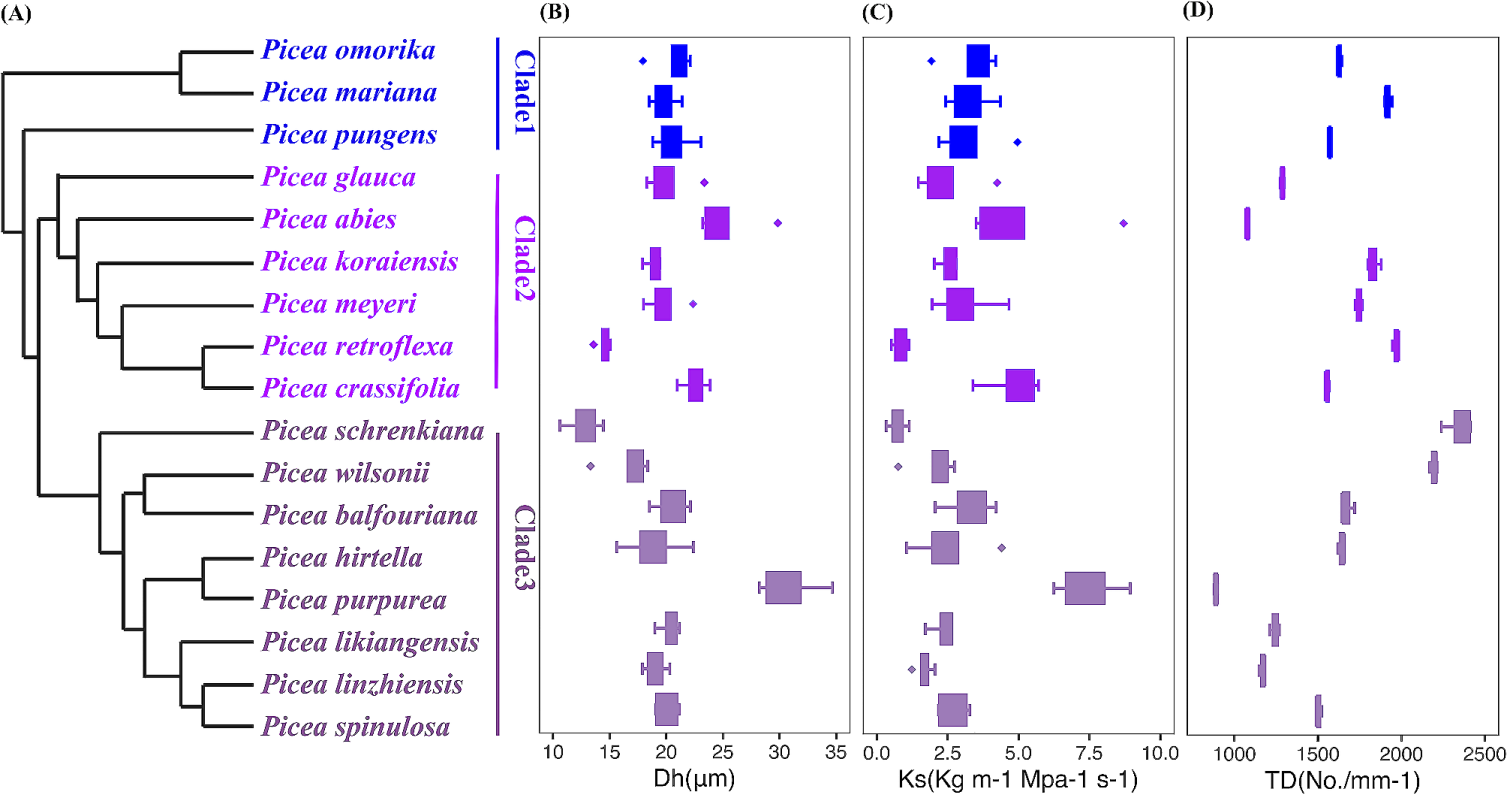
An unpartitioned ML tree of *Picea* reconstructed from the concatenated alignment of CDS (1st + 2nd) sequences of 1,141 OGs, labels to the right of the tree indicate sections (black line) following the accepted taxonomy for the genus (**A**). The mean and variance are shown with box and whisker plots for each species for mean tracheid hydraulic diameter (µm; **B**), theoretical hydraulic conductivity (kg m^− 1^ MPa^− 1^ S^− 1^; **C**), and the number of tracheids (TD).

We used Blomberg’s K metrics and Pagel’s λ to determine whether the *K*_s_ and related pit and tracheid traits of *Picea* are phylogenetically conserved. The hydraulic traits *K*_s_ and most features related to pit size and sealing exhibited no significant phylogenetic signals (Table 3; Fig. 6). Finally, the CWR, TSR, and TD showed low-phylogenetic signals based on Biomberg’s K and Pagel’s λ (Blomberg’s *P*_CWR_=0.03, *P*_TSR_=0.07, *P*_TD_=0.07). The anatomical traits of different phylogenetic groups (DPA, TO, MF, VE, and RH) were also significantly different but barely affected by phylogenetics.

**Table 3:** Differences in hydraulics and underlying traits among the three phylogenetic groups

Traits		Phylogenetic test				*F*	*P*	Clade 1	Clade 2	Clade 3
		Blomberg’s		Pagel’s λ						
		*K*	*P*	*λ*	*P*					
Mechanics	**CWR**	0.92	**0.03**	0.99	0.35	0.54	0.59	43.96(1.76)	42.33(2.14)	42.17(3.09)
	DWT	0.41	0.98	< 1	1	0.03	0.96	4.53(0.66)	4.58(0.20)	4.54(0.20)
	FL	0.756	0.142	< 1	1	0.06	0.93	1273(63.9)	1291(127.7)	1310(197.9)
	RH	0.636	0.376	< 1	1	3.99	**0.04**	16.75(0.27)	18.263(0.86)	17.44(0.82)
	NR	0.676	0.259	< 1	1	0.29	0.74	6.17(0.96)	6.09(0.81)	6.41(0.73)
Tracheid traits	D_r	0.58	0.54	< 1	1	0.19	0.82	20.80(2.62)	20.90(1.55)	20.25(2.15)
	LD_r	0.54	0.67	< 1	1	0.43	0.65	16.36(2.30)	16.70(2.06)	15.67(2.01)
	D_c	0.58	0.54	< 1	1	1.39	0.28	19.24(2.40)	18.16(0.66)	19.33(1.30)
	LD_c	0.54	0.66	< 1	1	1.80	0.20	14.93(1.81)	13.81(0.67)	14.98(1.27)
	**TSR**	0.81	**0.07**	0.99	0.48	0.17	0.84	0.59(0.02)	0.61(0.05)	0.60(0.06)
	Dh	0.74	0.18	0.99	0.56	0.01	0.98	20.39(0.51)	20.18(3.58)	19.88(5.07)
	Ks	0.67	0.32	0.99	0.41	0.01	0.98	3.30(0.09)	3.12(1.58)	3.03(2.50)
	**TD**	0.82	**0.07**	0.50	0.43	0.10	0.89	1700(175)	1566(338)	1587(511)
Pit traits	DPM	0.63	0.38	< 1	1	0.29	0.74	12.04(0.25)	12.31(0.93)	11.97(0.83)
	DPA	0.70	0.18	0.74	0.60	3.12	**0.07**	3.15(0.37)	3.83(0.47)	3.46(0.34)
	DT	0.67	0.25	0.13	0.84	1.38	0.28	6.11(0.33)	6.65(0.62)	6.30(0.45)
	TO	0.76	0.14	0.90	0.50	0.54	**0.01**	0.48(0.03)	0.42(0.02)	0.45(0.02)
	MF	0.77	0.12	0.91	0.44	4.83	**0.02**	0.49(0.01)	0.45(0.01)	0.47(0.01)
	VE	0.75	0.14	0.90	0.53	5.40	**0.01**	0.24(0.02)	0.19(0.01)	0.21(0.01)

Phylogenetic test for traits and one-way ANOVA with Tukey’s post-hoc test. Significantly different groups (*P* < 0.05) are indicated by other letters. Bold font represents significant values

### Origin legacy effect on key functional traits

Pearson correlations between the traits and origin climate indicated that most traits were strongly correlated with longitude (*P* < 0.001, Table 4) and not with elevation or mean temperature during the wettest quarter. Different tissue structures were affected to different degrees, with pit traits being more susceptible to the effect of the origin climate, followed by the PCA score, mechanical characteristics, and tracheid traits, which barely changed with climate change. As precipitation and drought index increased, the pit membrane diameter, torus diameter, and pit aperture diameter decreased, prompting pits to alter their flexibility to cope with the stress of reduced precipitation. Species from areas with higher temperatures tended to have smaller pits, larger tracheids, and a stronger anti-embolism ability. Furthermore, species from areas with less precipitation had larger pits and smaller tracheids, which increased the diameter of the pits to improve water transport efficiency and maintain normal water transport in the xylem. Mechanical properties were also affected by a moderate provenance climate, and FL was shortened with decreased precipitation and drought index. Only the LD_r slightly increased with increasing APRE and AI among the tracheid traits. Overall, the anatomical characteristics of the xylem of the *Picea* species and PCA scores were greatly affected by longitude, AMT, APRE, PREDQ, and AI (*P* < 0.05, Table 4).

**Table 4:** Pearson correlations between original climate and geographic data, anatomical characteristics of xylem, and the first two PCA scores from Fig. 2B

Traits		Origin Climate									
		Ele	Lat	Lon	AMT	MTWQ	MTDQ	APRE	PREWQ	PREDQ	AI
Mechanics	CWR	-0.26	0.15	-0.01	0.51*	0.40	0.17	0.00	-0.39	0.23	-0.16
	DWT	0.10	0.22	-0.51*	0.40	0.34	0.26	0.22	0.14	0.16	0.04
	FL	0.27	-0.14	-0.30	-0.17	-0.45	0.30	0.44	0.67**	0.12	0.48*
	RH	0.41	-0.32	0.59**	-0.44	-0.10	-0.30	-0.30	0.20	-0.47*	-0.19
	NR	0.00	-0.25	0.14	-0.13	0.04	-0.17	-0.12	-0.41	0.02	-0.13
Tracheid traits	D_r	0.03	0.30	-0.61**	0.04	-0.05	0.19	0.44	0.40	0.28	0.40
	LD_r	0.00	0.36	-0.63**	0.01	-0.16	0.25	0.46*	0.43	0.29	0.48*
	D_c	0.26	0.09	-0.65**	0.24	0.05	0.31	0.31	0.29	0.17	0.12
	LD_c	0.39	-0.06	-0.56**	0.17	-0.09	0.37	0.31	0.34	0.14	0.11
	TSR	-0.16	0.06	0.24	0.27	0.43	-0.11	-0.33	-0.44	-0.13	-0.35
	Dh	0.12	0.10	-0.18	-0.23	-0.35	0.09	0.26	0.29	0.13	0.32
	Ks	0.14	0.07	-0.02	-0.24	-0.30	0.00	0.10	0.12	0.03	0.17
	TD	-0.15	-0.01	0.37	0.15	0.31	-0.20	-0.38	-0.46	-0.17	-0.44
Pit traits	DPM	-0.01	-0.12	0.38	0.05	0.42	-0.38	-0.41	-0.16	-0.34	-0.51*
	DPA	0.24	-0.39	0.80***	-0.25	0.30	-0.56**	-0.61**	-0.02	-0.65**	-0.58**
	DT	0.13	-0.28	0.65**	-0.12	0.39	-0.52**	-0.56**	-0.10	-0.55**	-0.59**
	TO	-0.35	0.46*	-0.78***	0.40	-0.05	0.46*	0.52**	-0.08	0.63**	0.40
	MF	-0.35	0.46*	-0.79***	0.38	-0.07	0.46	0.52**	-0.08	0.63**	0.41
	VE	-0.34	0.46*	-0.78***	0.41	-0.05	0.46*	0.52**	-0.08	0.64**	0.40
PCA	PC1	-0.05	-0.23	0.69**	-0.06	0.29	-0.39	-0.53**	-0.35	-0.39	-0.49*
	PC2	0.40	-0.37	0.55**	-0.54**	-0.19	-0.38	-0.28	0.37	-0.52**	-0.14

The bold coefficients indicate *P* < 0.1, *; *P* < 0.1, **; *P* < 0.05, ***; *P* < 0.001. For Origin Climate abbreviations, see Table S1

## Discussion

### Variations in growth traits and xylem anatomical traits

Quantitative analysis of interspecific differences in xylem anatomy and growth traits is the basis for predicting tree response and viability to climate change [36, 37]. Tree height and basal diameter at 6 years of age of the 17 spruce species showed extremely significant interspecific differences, consistent with the previous study with the same difference in trends [29]. Large interspecific variation may be the result of long-term evolutionary adaptation to the environment of the origin, and tree height and diameter at breast height have the same variation trend among species, implying that the traits were under the same genetic control [38]. *P. abies, P. pungens, P. likiangensis*, and *P. linzhiensis* exhibited the fastest and most stable growth rates at both 6 and 9 years of age. The rapid growth rate showed that these species fit the current living environment. Since long-term living species in extreme climates tend to have higher adaptability and resilience [39], the four species that came from extreme temperatures (areas with higher or lower altitudes, or high average annual precipitation, or high drought index) would possess the greatest biomass in a controlled environment [40].

Interspecific growth differences explain the altering xylem structure. To be in a favourable position in the process of evolution and succession, the anatomical characteristics of plant stems showed obvious differences according to environmental changes. There are large interspecies differences in xylem mechanical traits, tracheid traits, and pit traits [15, 41], consistent with the results of our study, indicating that the 17 spruce species have different embolism resistances and hydraulic transport capacities [42]. Plants with smaller vessels or tracheid diameters, pit diameters, and pit openings are generally believed to be more drought-resistant [43]. Among the 17 spruce species, *P. hirtella, P. schrenkiana, P. meyeri*, and *P. mariana* had smaller tracheids, indicating that these tree species were more resistant to drought [15, 39]. Although significant differences in xylem anatomy between species were revealed at the family level, the materials in this study came from the same garden, thus eliminating the impact of planting environment on wood anatomical properties. For Zhang’s study, xylem anatomy differences were investigated among nine deciduous broad-leaved tree species from six families, including *Fraxinus mandshurica*, *Quercus mongolica*, *Ulmus macrocarp*, etc. However, the experimental places were along the slope of a river valley, ignoring the potential impact of the planting site environment on xylem anatomical traits [44].

### Trade-offs among wood anatomical traits

Plants exhibit different return-on-investment strategies to adapt to climate; thus, functional traits and their associations may reflect an organism’s response to the climate. The synergistic and trade-off relationships between functional characteristics could have different patterns and mechanisms. Our research also contributed to the trait relationships of 18 coniferous tree species from 4 families. Surprisingly, our findings align with Pittermann’s conclusions. *K*_s_ exhibited a significant negative correlation with TSR, CWR, and TD, suggesting that an increase in the number of tracheids per unit area would lead to a decrease in tracheid diameter rather than wall thickness, indicating a trade-off between strength and efficiency [45]. A greater wall thickness to tracheid diameter ratio reinforces the mechanical resistance against tracheid implosion because of increased negative pressure during drought [46]; however, *K*_s_ was positively correlated with FL, D_r, and LD_r. Thus, larger hydraulic diameters tend to have larger tracheids, whereas the TD per unit area decreases. When plants are under drought stress, the TD per unit area decreases, and plants increase *D*_h_ by increasing the size of tracheids. Furthermore, to safeguard the hydraulic strategy of the plant body, a larger FL and smaller CWR and TSR are required to maintain the supporting role of the xylem and maintain a stable water transport mechanism.

The trade-off between hydraulic efficiency and mechanical strength was also existed in the *picea* genus in our study (Fig. 4), which confirmed previous findings [19, 46] and contradicted others; the evidence for a trade-off between mechanical strength and hydraulic efficiency remains ambiguous. Fan et al. [47] have shown that species with larger xylem vessel sizes had higher high theoretical hydraulic conductivity (*K*_s_). Meanwhile, higher xylem hydraulic efficiency can only be achieved by the cost of reduced WD and mechanical strength. Those earlier descriptions of such a trade-off probably resulted because the conflicting structural requirements in xylem design would lead to a “trade-off triangle” among mechanical strength, conductive efficiency, and resistance to embolism [42]. The above conclusions reflect the trade-off strategies of resource investment and income when species with different functional traits respond to the external environment and reflect the internal mechanism of plant niche differentiation and species coexistence [42].

### Effects of anatomical traits on hydraulic conductivity and growth

To explore how xylem anatomical traits regulate growth, we performed a regression analysis and showed that tree height was negatively correlated with pit membrane diameter (DPM) and TSR and positively correlated with the *D*_h_ and DPA (Fig. 5E and H, Fig. S1C). This was similar to a study on *P. cembra* and *P. abies* along an elevational transects [24], contrary to the results for Norway Spruce [8]. In our study, the size of the tracheids significantly affected hydraulic conductivity (Fig. 5C and D), consistent with a previous study [15], as water transport through wide structures reduces friction between water and plant cells and facilitates water flow [48]. Also, hydraulic conductivity was significantly negatively related to tracheid CWT, TSR, and Pits (Fig. 5A and B; Fig. S3C), consistent with the results reported by Song [15]. Song and Hacke suggested that thicker cell walls may increase the hydraulic path length within the pits and, therefore, the pit aperture resistance, which would reduce *K*_s_ and the lumen area available for fluid flow [49, 50]. In SEM (Fig. 4C), the results indicated that D_r, TSR, and DWT influenced *K*_s_ through indirect pathways. Our conclusion was based only on the theoretical *K*_s_ calculated using formulas. Therefore, in subsequent studies, we systematically combined the actual measured *K*_s_ values to quantify the impact of anatomical features on *K*_s_.

### Relations of wood anatomical traits to phylogeny and original environment

We found no phylogenetic signals for the traits except for CWR, TSR, and TD, which have weak phylogenetic signals (Table 3; Fig. 6), consistent with the previous findings on oaks [50] and 23 *Picea* species [30]. This suggests a strong divergent selection in the anatomical characteristics that define wood anatomies in the spruce genus, regardless of phylogenetic proximity. We propose that the non-conservatism of these traits is attributed to environmental filtering, which may select taxa that are phylogenetically more distant but functionally more similar [51], implying that these traits have large variations in the evolutionary process and are easily affected by the environment. Traits are not conserved, meaning that these traits allow species to radiate into different habitats, confirming the evolutionary method of radiation differentiation in the *Picea* genus [52]. The phylogeny of the xylem anatomy is influenced by the methodology used for its determination, the number and the size of taxa studied (size of the phylogeny), and the settings in which the plants grow (field vs. common garden) [53]. Traits may have significant phylogenetic signals in studies with larger pedigrees or ranges, which are not obvious in studies with smaller pedigrees [15, 54]. Strong phylogenetic signals were detected in 4 families with 12 genera in seagrasses and were found in 4 genera with 27 species [55, 56]. In contrast to the subjects of the aforementioned studies, our materials are exclusively from the *Picea* genus and have a smaller pedigree, resulting in no strong phylogenetic signal being detected.

In addition, our study revealed that many xylem properties within the *Picea* genus showed a correlation with the climatic niche of the species, which is consistent with the findings of Song [57]. Longitude significantly affects the anatomical structure of the xylem. We speculated that it could affect photoperiod, thereby regulating the structure of the xylem. In the common garden of Tianshui, the relationship between stem traits and species climate of origin demonstrated that climate strongly affected xylem structure, which minimized environmental variation and phenotypic plasticity. This yields valuable insights into the genetic constraints of plant traits [6]. In the present study, we provide evidence of strong genetic control over the development of these traits. The genetic constraints of pit traits indicated a limited capacity for short-term acclimation to novel climate scenarios, which is supported by recent studies showing limited intraspecific variation in cavitation resistance across aridity gradients. In this study, compared with Tianshui, tree species from areas at lower altitudes with greater precipitation had smaller DPA and DT, such as *P. glauca, P. pungens, P. abies* and *P. omorika*, which might have stronger embolism resistance. We conclude that the closely joined evolution of high embolism resistance with a small pit size, higher VE, and pit aperture resistance has enabled conifer species to be highly resistant to drought, with tree species originating from low average annual temperatures and little precipitation planted in Tianshui. To cope with the drought stress caused by increasing temperatures, they have larger tracheids, such as *P. crassifolia, P. koraiensis*, and *P. hirtella*, to improve water use efficiency and compensate for the evaporation caused by rising temperatures. We also found that tree species from high altitudes, high average annual temperatures, and high precipitation, such as *P. abies, P. balfouriana, P. crassifolia, P. mariana*, and *P. omorika*, had larger *K*_s_ to maintain the xylem’s demand for water, and their growth rate was relatively high. Since their introduction, different spruce species have shown different survival strategies. Therefore, in the short term after introduction, compared with the original growth environment, the species suffered from water and drought stress, promoting plants to cope by changing effective trait combinations with high adaptability [17].

## Conclusions

The growth traits and functional traits of the stem xylem of the *Picea genus* exhibited varying degrees of trait variation within and among species. Moreover, mechanical traits, pit traits, and tracheid characteristics were intricately interconnected and regulated the trait development of stems. Most of the characteristics had no or low phylogenetic signals, and variation in most of the traits was affected by the origin climate, especially the longitude, precipitation, temperature, and drought index. This study filled research gaps on the interspecific differences in the anatomical structure of the stem xylem of the *Picea genus*, especially on pits and played a certain guiding role in the cross-regional introduction of the *Picea genus*. When introducing spruce, the difference between the planting place and the place of origin should be considered, and the precipitation, temperature, and drought index should be as consistent as possible with the place of origin, to maximize its production and ecological benefits. In addition, multi-site experiments are warranted to observe the species variation of these traits over a long period and explore the mechanism underlying species adaptation to the environment, which is key to understanding how ecosystems regulate important functions to maintain survival and growth strategies in the context of climate change.

### Electronic supplementary material

Below is the link to the electronic supplementary material.

Supplementary Material 1

Supplementary Material 2

Supplementary Material 3

Supplementary Material 4

Supplementary Material 5

## Data Availability

The datasets generated and/or analysed during the current study are available from the corresponding author upon reasonable request.
