# Supplementary figures and images for: Climate of origin shapes variations in wood anatomical properties of 17 Picea species
Source: BMC Plant Biol. 2024 May 17;24:414. doi: 10.1186/s12870-024-05103-7 (PMC11100223; doi:10.1186/s12870-024-05103-7)

**Fig. S1** Bivariate relationships between H6 and underlying properties (A–O) for 17 *Picea* species.


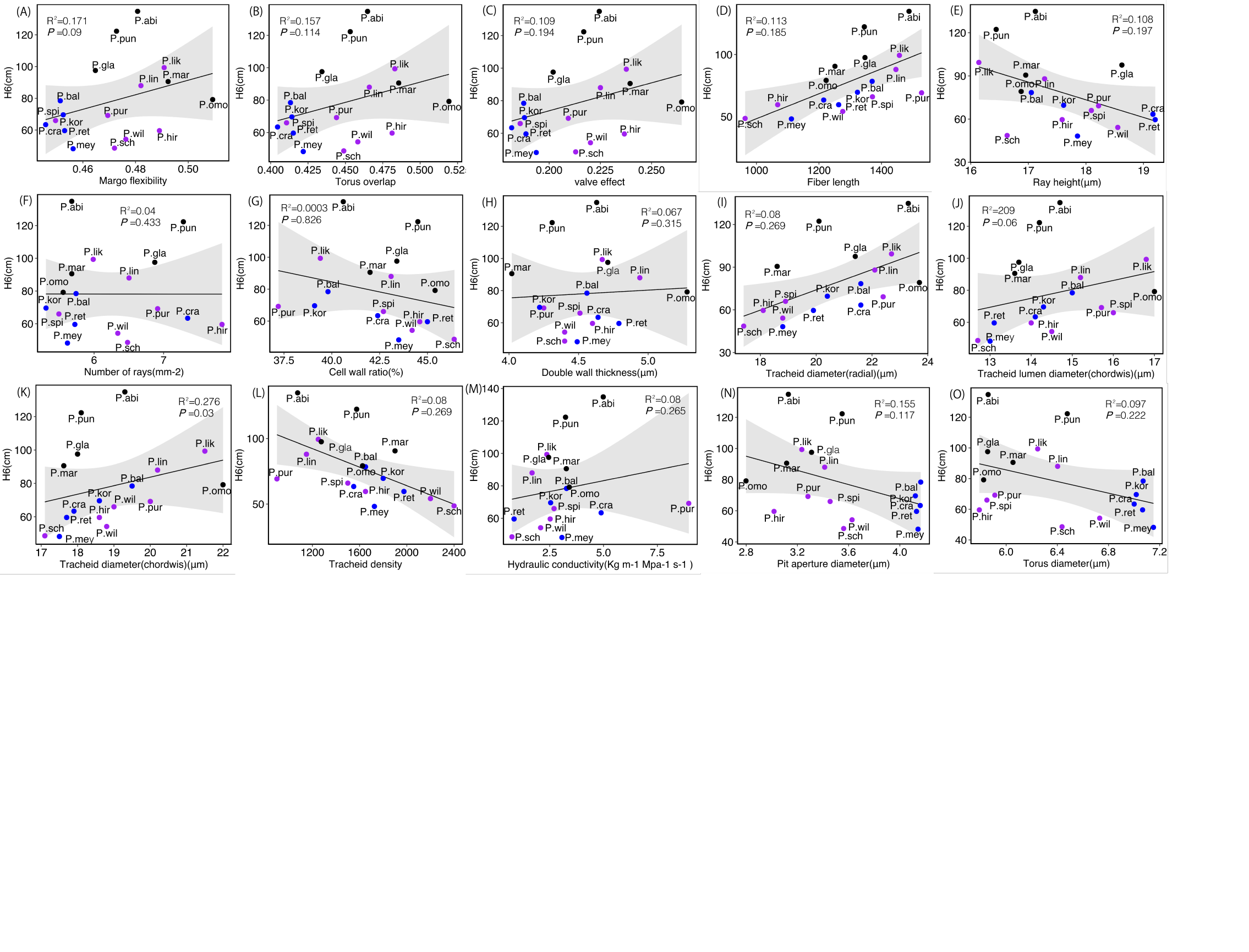

Supplement: Supplementary file 3 — Supplementary Material 3 [file 12870_2024_5103_MOESM3_ESM.docx]

**Fig. S2** Bivariate relationships between BD6 and underlying properties (A–O) for 17 *Picea* species.


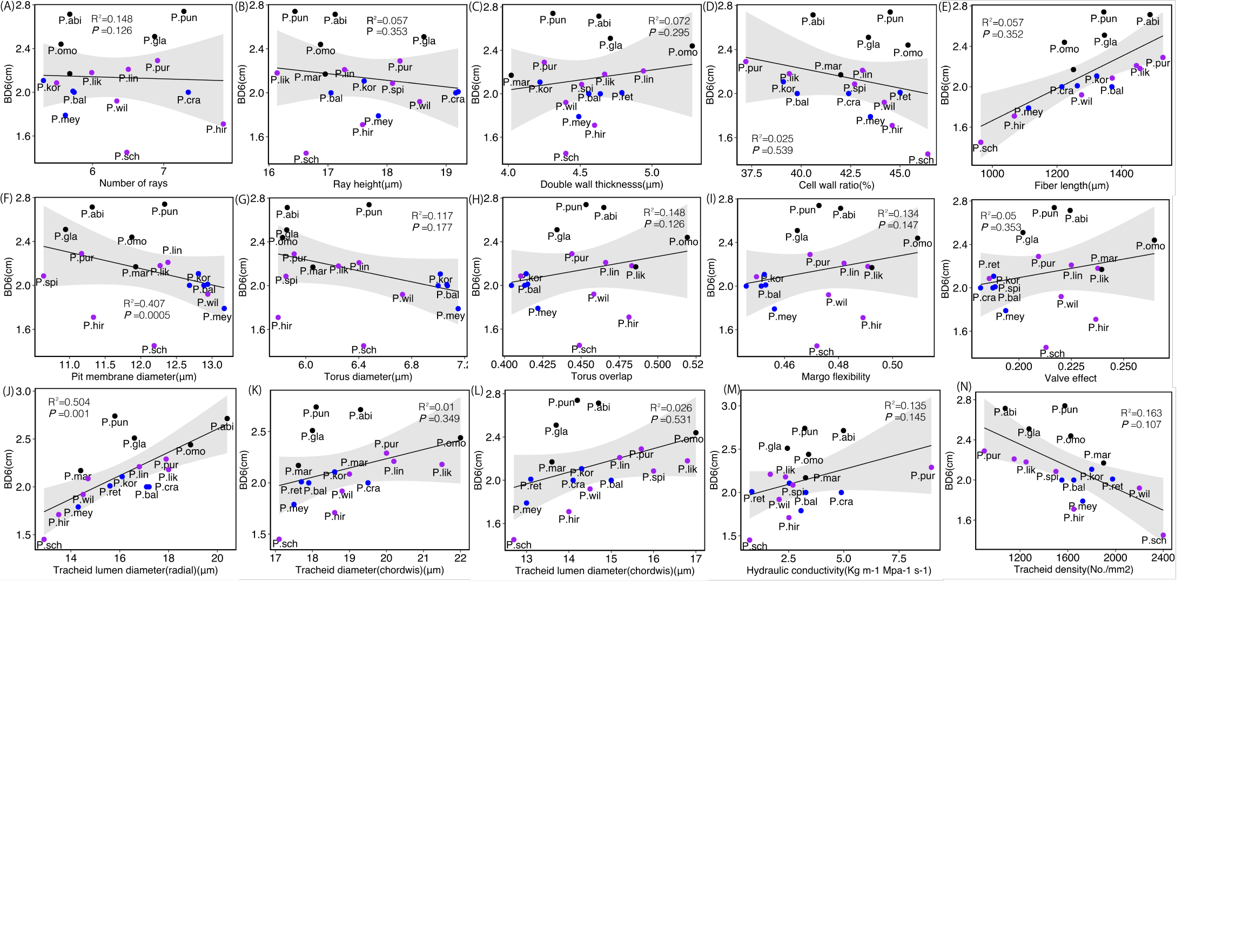

Supplement: Supplementary file 4 — Supplementary Material 4 [file 12870_2024_5103_MOESM4_ESM.docx]

**Fig. S3** Bivariate relationships between Ks and underlying properties (A–N) for 17 *Picea* species.


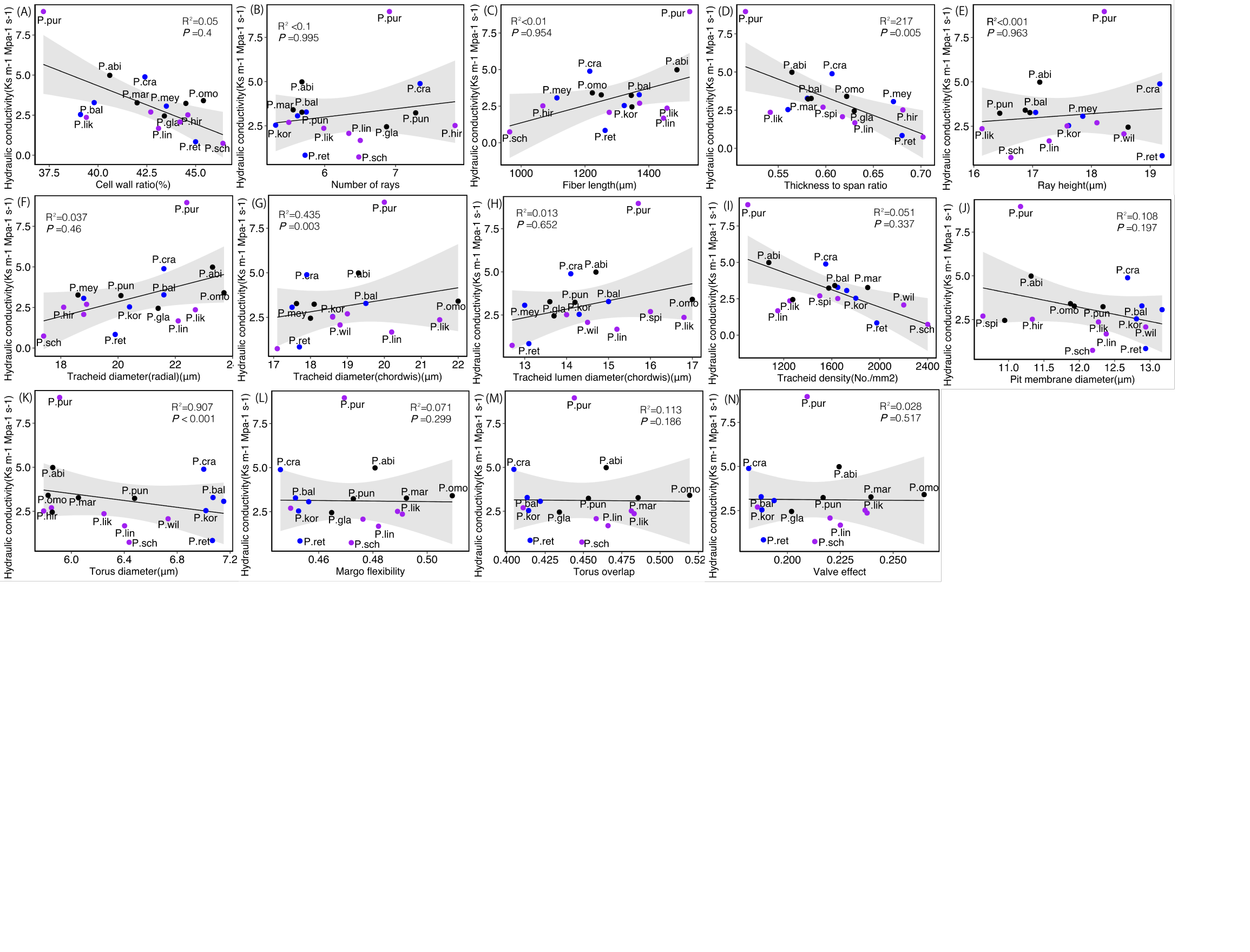

Supplement: Supplementary file 5 — Supplementary Material 5 [file 12870_2024_5103_MOESM5_ESM.docx]
